# Supplementary material for: Learning curve and surgical outcome of robotic assisted colorectal surgery with ERAS program
Source: Sci Rep. 2022 Nov 29;12:20566. doi: 10.1038/s41598-022-24665-w (PMC9709162; doi:10.1038/s41598-022-24665-w)
Supplement: Supplementary file 1 — Supplementary Information 1. [file 41598_2022_24665_MOESM1_ESM.zip › LSC Raw data-Ñ[▒K/101/N_ñw╝╨░O▒Kñσ.pdf]

W52 / W52

臺中榮民總醫院
手術室護理記錄

[redacted]

基本資料

- 室別： 開刀房-06室，手術科別： CRS，手術類別： 常規，日期： 2020/10/29
- 進入時間： 1205 ； 麻醉開始時間： 1210 ； Time Out時間： 1330 ； 劃刀時間： 1331 ； 手術結束時間： 1730 送出時間： 1805 ；
- 麻醉方式： GE

  診斷： Malignant tumor of T-colon,PROVED

  手術名稱： 1.Laparoscopic right colectomy and anastomosis2.Left hemicolectomy with LN dissection

  手術局部用藥： 無

  術前預防性抗生素：
  - 抗生素名稱一： Veterin 1GM IVD/由麻醉護理師執行
  - 時間： 1315
  - 上次簽章: 陳祐溥

  ，追加預防性抗生素：
  - 抗生素名稱一： Veterin 1GM IVD/由麻醉護理師執行
  - 時間： 1700
  - 上次簽章: 陳映庭
- 手術醫師： 蔣鋒帆，陳映菁，林昌霖，陳智翔
- 麻醉醫師： VS 王賜洲
-
  - 刷手護士A ： 陳映庭，時間： 1205，接替刷手一： 何佳青，時間： 1230，接替刷手二： 陳秋誼，時間： 1500
  - 流動護士A ： 陳秋誼，時間： 1205，接替流動一： 陳祐溥，時間： 1230，接替流動二： 陳映庭，時間： 1500
- 更多接替及流動護士： 接替流動三： 高子貽，時間： 1730

皮膚狀況

A-V Shunt： 無

術前： 完整

記錄者： 上次簽章: 陳秋誼

術後： 完整

記錄者： 上次簽章: 高子貽

手術準備

手術臥式 單一手術臥式

-
  - 手術臥式： 截石臥式
  - 使用附件： 溫毯 38 ℃，矽膠枕頭，腳架，其它： 頭架
  - 皮膚準備： 10%Aq Betadine，Hibiscrub，2% Chlorhexidine
  - 電燒： 使用電燒: 電極片位置： 其它： 迴路電擊墊，記錄者： 上次簽章: 陳秋誼

止血帶T： 無

植入物

- 植入物名稱/號碼/數量/部位： COVIDEN ENDO GIA 80mm-3.8mm x2/大腸

，記錄者： 上次簽章: 陳映庭

標本送檢

- 細菌培養 無
- 細胞學 無
- 冰凍切片 無
- 病理標本

  組織名稱一： A to D colon

- 截肢 無

標本交付 標本： 1 袋 ， 標本： 0 盒 ， 標本： 1 瓶 ， 細胞學： 0 管 ， Culture Tube： 0 管 ， 被交付醫師： CR 陳映菁 ，

- 記錄者： 上次簽章: 陳映庭
- 時間： 1720

傷口術後敷料

傷口敷料： 傷口敷料： 無

- 請於圖上標示部位：

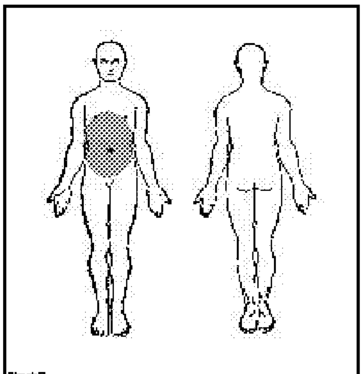

- 傷口敷料： 塗傷口凝膠及軟性敷料覆蓋

導管

導管	天數	導管資訊	插入日期	插入時間	更換日期	拔除日期	拔除時間	拔除原因	預計拔除/更換	預計拔除/更換日期
Foley(1)	1	號碼:16 Fr.; 部位:膀胱;	2020/10/29	1250						
Double Lumen CVC(1)	1	號碼:8 Fr.; 深度:Fix 15 cm; 部位:jugular (右); 象限:RU;	2020/10/29	1245						
A-line (1)	1	號碼:20 G; 部位:左手;	2020/10/29	1240						

用物計數

- 手術中出血量： 200 c.c.
- 手術中尿量： 250 c.c.
-
  - 其它： 無

用物計數： 多切口同時縫合

- 1.器械： 正確 ， 2.縫針： 正確 ， 3.刀片 ： 正確 ， 4.紗布： 正確 ， 5.腹部墊： 未使用 ， 6.紗球： 未使用 ， 7.PEANUTS ： 未使用 ， 8.紗條： 未使用 ， 9.絲帶： 未使用 ， 10.針頭： 未使用 ， 11.Vessel loop： 未使用 ， 12.Boots： 未使用 ， 13.Jelco： 未使用 ， 14.棉條： 未使用 ，
  - 第一次：
    - 刷手護士 ： 陳秋誼
    - 流動護士： 上次簽章: 陳映庭
    - 時間： 1420

  ,
  - 第二次：
    - 刷手護士 ： 陳秋誼
    - 流動護士： 上次簽章: 陳映庭
    - 時間： 1440

  ,
  - 第三次：
    - 刷手護士 ： 陳秋誼
    - 流動護士： 上次簽章: 陳映庭
    - 時間： 1500

備註

備註： CRS 腹腔鏡5號。

- 記錄者： 上次簽章: 陳秋誼
- 時間： 1215

填寫護理人員 高子貽

正式摘要

	W52 / W52

doctorRank:B
